# Cross-Modal Interactions and Movement-Related Tactile Gating: The Role of Vision

**DOI:** 10.3390/brainsci15030288

**Published:** 2025-03-08

**Authors:** Maria Casado-Palacios, Alessia Tonelli, Claudio Campus, Monica Gori

**Affiliations:** 1 DIBRIS—Department of Informatics, Bioengineering, Robotics and Systems Engineering, University of Genoa, 16145 Genoa, Italy; 2 UVIP—Unit for Visually Impaired People, Italian Institute of Technology, 16152 Genoa, Italy; alessia.tonelli@iit.it (A.T.); claudio.campus@iit.it (C.C.); monica.gori@iit.it (M.G.)

**Keywords:** active touch, audio, blindness, cross-modal processing

## Abstract

Background: When engaging with the environment, multisensory cues interact and are integrated to create a coherent representation of the world around us, a process that has been suggested to be affected by the lack of visual feedback in blind individuals. In addition, the presence of voluntary movement can be responsible for suppressing somatosensory information processed by the cortex, which might lead to a worse encoding of tactile information. Objectives: In this work, we aim to explore how cross-modal interaction can be affected by active movements and the role of vision in this process. Methods: To this end, we measured the precision of 18 blind individuals and 18 age-matched sighted controls in a velocity discrimination task. The participants were instructed to detect the faster stimulus between a sequence of two in both passive and active touch conditions. The sensory stimulation could be either just tactile or audio–tactile, where a non-informative sound co-occurred with the tactile stimulation. The measure of precision was obtained by computing the just noticeable difference (JND) of each participant. Results: The results show worse precision with the audio–tactile sensory stimulation in the active condition for the sighted group (*p* = 0.046) but not for the blind one (*p* = 0.513). For blind participants, only the movement itself had an effect. Conclusions: For sighted individuals, the presence of noise from active touch made them vulnerable to auditory interference. However, the blind group exhibited less sensory interaction, experiencing only the detrimental effect of movement. Our work should be considered when developing next-generation haptic devices.

## 1. Introduction

When we interact with our environment, we are flooded with information from multiple sensory channels, such as drumming our fingers on the table and producing a sound or moving our feet in the sea and seeing and hearing them splash. In these moments, our brain integrates the information from the multiple senses into one unified percept to provide us with a coherent interpretation of the world. Nevertheless, when we receive multisensory information, one sense can dominate our perception due to its stronger reliability [1,2]. In contrast, cues can be integrated to infer the most likely interpretation of the sensory input when we face sensory ambiguity [1]. Many studies report a perceptual benefit when multisensory integration is triggered. For instance, reduced reaction times have been found in the presence of redundant multisensory feedback [3]. Nonetheless, multisensory interaction can also be detrimental when conflicting information from one sensory modality affects the perception of a second modality [4] or acts as a distractor [5].

Interestingly, it has been suggested that multisensory processing is a late development achievement, as children do not show an adult-like integration until 8–10 years of age. Until this time, one sensory modality always dominates over the other [6,7]. For instance, in a visuo-haptic experiment, it has been suggested that there is a unisensory dominance in younger children that changed depending on the specific tasks; in the discrimination of orientation, vision dominated the perception, while in the discrimination of size, touch had a predominant role [6]. One possible explanation for this process may lie in the differing maturation timelines of each sensory modality. The progressive development of unisensory systems may facilitate multisensory processing by enabling the brain to identify and integrate statistical patterns across sensory inputs in daily life [8]. In this line, the cross-modal calibration theory suggests that before effective multisensory processing can occur, continuous recalibration between perceptual systems is necessary through cross-sensory comparison. During this process, the sense that provides the most reliable information calibrates the others [6], leading to optimal integration by allowing a proper weighting of the sensory signals available [9,10]. Consequently, according to this theory, multisensory processing might be compromised by the absence of a sensory system. For example, in the perception of space, the visual modality conveys the most reliable information [11]. What, then, happens when visual calibration is missing? The study of multisensory perception in blindness allows us to understand the role of vision in this process. Previous research shows that the absence of visual input can indeed impact multisensory processing, as there is a reduced interaction between tactile and auditory feedback in the perception of space in this population [12,13]. In particular, Hötting and Röder [12] reported that congenitally blind participants were less affected by task-irrelevant stimuli than their sighted counterparts when performing a tactile–auditory illusion task. Similarly, Occelli and colleagues [13] obtained the same results using a ventriloquist paradigm. This task consists of the biased localization of one stimulus perceived from one sensory modality by another simultaneous and spatially discrepant stimulus conveyed by another modality. These researchers discussed the reduced ventriloquist effect found in the congenitally blind group as evidence of the diminished audio–tactile interaction caused by a lack of visual input early in life [13]. Previous research has indicated that this difference stems from the absence of an external coordinate system in blind individuals, with vision being crucial for its development. However, more recent studies imply that the variations in cross-modal interactions between sighted and blind individuals may instead result from differences in the weighting of external versus internal coordinate systems [14,15]. These findings suggest that, while blind individuals can use an external coordinate system under certain conditions, such as by the use of instructions designed to encourage its use (i.e., discrimination between left vs. right hemispace), they typically assign less weight to this information, with its use not being automatic [15]. Due to this, when blind individuals need to combine audio, mostly coded in an external reference frame, and tactile feedback, coded in an internal one, the lack of a common frame of reference might create difficulties with its interactions [16].

Nevertheless, in multisensory research, the interaction between audio and touch is an underexplored topic, even in the sighted population. It is especially true when somatosensory feedback is generated during active touch. Active touch, which is defined as the sensation of external textures elicited during voluntary human movement, is argued to be the most natural form of touch. It differs from passive tactile perception not just in terms of the mechanoreceptors activated and the processes involved [17,18] but also because the movement generated during active touch might add an obstacle to the processing of tactile inputs in the somatosensory cortex. A self-elicited movement can gate, or diminish, the transmission of tactile signals to the parietal centers involved in their decoding [19,20]. This phenomenon is known as movement-related tactile gating [17,18,19]. Surprisingly, although the importance of movement during the tactile experience has been highlighted [21,22], and the ongoing efforts to develop multisensory haptic solutions designed for both the general population and blind individuals, the impact of this phenomenon on multisensory processing and the role of vision in it have not yet been traced. Therefore, in this study, we explored whether cross-sensory interaction can be affected by self-elicited movement and how vision can modulate this effect. To this end, we asked sighted and blind participants to identify the faster stimulus between two velocities using dynamic stimuli. We hypothesized (1) that there would be a non-significant difference between the unimodal and the bimodal audio–tactile stimulations for both groups in the passive condition as well as (2) in the active condition in blind participants. In contrast, we expected (3) significantly different performances between unimodal tactile and bimodal audio–tactile stimulations during active touch in the sighted participants. (4) Additionally, we expect movement to cause a worse performance in blind individuals, regardless of the presence of auditory feedback.

## 2. Materials and Methods

### 2.1. Participants

This paper considered the number of participants and data from our previous experiment [23]. For the tactile conditions (passive and active), we used the same data from our previous study [23]. They served as baseline to define the participants’ ability to perceive speed tactually for this paper. In the present study, we recalled all participants included in the previous study to complete the audio–tactile conditions tested in the current experiment.

A total of 18 blind participants (10 women, mean age ± SD: 41.67 ± 11.9 years) and 18 age-matched sighted individuals (12 women; mean age ± SD: 35.11 ± 11.72) took part in our study (age difference not significant: t(34) = 1.67, *p* = 0.105). Table 1 summarizes the clinical information of the blind group. All subjects stated they had no history of sensory–motor (besides blindness) or cognitive deficits. The local health service’s ethics committee (Comitato Etico, ASL3 Genovese, Genoa, Italy) approved the research protocol (Comitato Etico Regione Liguria, Genoa, Italy; Prot. IIT_UVIP_COMP_2019 N. 02/2020, 4 July 2020), and the experiment was performed according to the Declaration of Helsinki. Informed consent was obtained from all participants.

### 2.2. Design and Procedure

**Figure 1:**
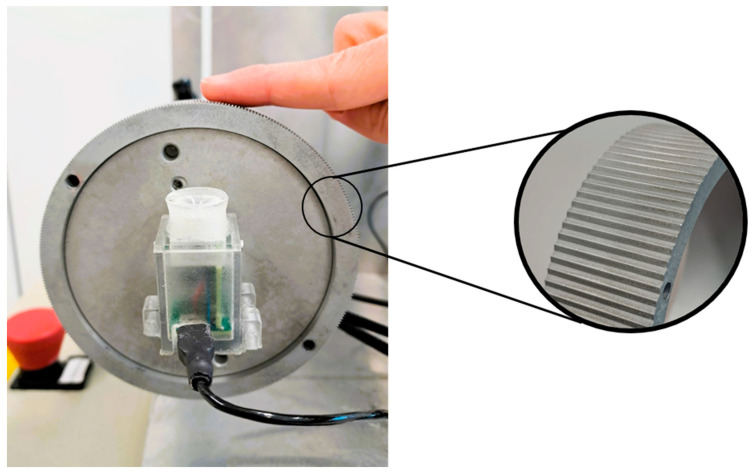
Experimental setup consisting of a 10 cycles/cm wheel with an auditory device attached to it. The position of the finger during the experimental task is also represented.

We used the same paradigm as in a previous study [23], asking the participants to discriminate between two velocities using dynamic stimuli to ensure more accurate perception [17]. We used tactile and auditory stimulation provided by two custom-made devices [24,25] (Figure 1). In particular, the tactile stimulation was delivered by a physical wheel (Figure 1) that contained a sinusoidal grating with a spatial frequency of 10 cycles/cm. The wheel moved in one direction (right or left randomly) during 500 ms and returned to the initial position in the following 500 ms. The speed of the movement varied between trials. The auditory stimulation was a 927Hz tone provided by the speaker of a MSI Caterpillar module [26] that was attached to the wheel (Figure 1). Both devices were operated using a custom MATLAB code (R2020b, The MathWorks, Natick, MA, USA). The sound duration of the sound matched the duration of the tactile stimulus.

The participants sat in a chair during the experiment with their body’s midline centered on the wheel. Figure 1 presents the wheel’s orientation and the finger’s position. Stimulation was applied to the fingertip of the index finger on the right hand, regardless of the manual dominance of the participant. Both the blind and sighted participants were blindfolded, removing the sight variable from both groups. During the task, the participants felt two consecutive speeds lasting one second each. The first was presented at a standard velocity of 3cm/s, while the second was presented at a random speed determined by the adaptive algorithm QUEST [27]; 500ms separated the two stimulations. The participants were required to detect the faster stimulus and report it verbally to the experimenter. We had two sensory stimulations: (1) tactile (T) and (2) audio–tactile (AT). The tactile sensory stimulus could be perceived either in (1) a passive condition, in which the finger was fixed in a specific position, or (2) an active condition, in which the finger would be actively moved in the opposite direction of the wheel’s movements by the participant. This movement was performed simultaneously with the wheel’s movement. The duration of the stimulation was constant regardless of the sensory stimulation provided and whether it was presented in the passive or active condition. The T passive and active conditions served as the baseline. This dataset was previously obtained and discussed in an earlier experiment [23]. AT passive and active were the new experimental conditions. By comparing the precision obtained with the bimodal audio–tactile sensory stimulation to the unimodal tactile in passive and active touch conditions, we aim to define how cross-modal interaction can be affected by passive and active tactile perception. In addition, including both sighted and blind participants in this study enables us to determine whether visual experience can alter this process. There were 30 trials per condition, resulting in a total of 120 trials: 60 from the previous unimodal experiment and 60 from the new bimodal experiment. In both unimodal and bimodal experiments, the passive and active conditions were randomized across participants. During the tactile-only sensory stimulus, a sound-isolating headphone was provided to eliminate the noise produced by the device to ensure the processing of only tactile information. Instructions were given to control the velocity of the finger [28]; participants were trained to move it at approximately 3 cm/s.

### 2.3. Data Analysis

We fitted the data using cumulative Gaussians for each condition. The best-fitting function’s mean and standard deviation yielded both the point of subject equality (PSE) and the just noticeable difference (JND) estimates. The standard errors for both estimates were obtained by bootstrapping [29]. In this case, we used the JND as a dependent variable, defined as the minimum velocity required to correctly identify the faster stimulus in 75% of the trials.

Then, we manually inspected each psychometric curve to identify those that were inverted (i.e., negative JND). An inverted psychometric function within our paradigm can only be attributed to poor participant performance, as it cannot be explained by the stimulation presented. To address this in the statistical analysis, we conservatively replaced the negative JND value with the maximum possible JND value within the experimental setup. In this study, the replacement value was set to 6 cm/s, corresponding to the maximum velocity that could be presented. Two blind individuals presented a negative JND. One showed an inversion in audio–tactile modality during active touch, and the other showed inversion for the same sensory modality in passive touch. Subsequently, we evaluated the presence of outliers for all the conditions, selecting those whose JND values were lower than the first quartile minus 3 times the interquartile range, as well as those greater than the third quartile and more than 3 times the interquartile range. One outlier was detected in the blind group for the audio–tactile in the passive condition. Consequently, 18 sighted and 17 blind participants were included in the analysis. Using the previously selected blind participants, we investigated whether differences in the onset time of blindness could affect their performance. A Pearson correlation analysis was computed. The “cor.test” function from the R package “stats” [30] was applied for this analysis using the JND obtained in each sensory stimulation (tactile and audio–tactile) and condition (passive and active) along with the blindness duration of the participants. This analysis aimed to guide our decision on group division. Specifically, whether to separate the blind participants into two groups (early and late blindness onset) or to merge them into a single group if performance was not affected by blindness duration.

#### Statistical Analysis

The next step was to assess the normality assumptions of each group and experimental condition using the Jarque Bera test (see Supplementary Materials). When the test result was significant, Wilcoxon tests were adopted using the “wilcox.test” function in R (package “stats”) [30] instead of *t* tests.

Finally, we performed the main analysis of our data. Given the results of our previous study [23] and the research questions of the current one, we made planned comparisons between our two sensory modalities (tactile and audio–tactile) separately for passive and active touch.

To further support our results, we computed the delta value (SensoryDelta) from the bimodal (audio–tactile) and the unimodal (tactile) modalities by subtracting the JND from the second to the first (1), and then, we performed planned comparisons against zero. This final step was repeated for the delta value (ConditionDelta) from the active and passive perceptions, again by subtracting the JND from the second condition to the first one (2), to explore the implications of the movement itself for our two sensory conditions.

(1) $${∆}_{SensoryDelta}={JND}_{AT}-{JND}_{T}$$

(2) $${∆}_{ConditionDelta}={JND}_{active}-{JND}_{passive}$$

## 3. Results

We found no correlations between the JND value and the blindness duration for the passive condition through the unimodal tactile stimulation (r = 0.04, *p* = 0.8785) or the bimodal audio–tactile one (r = −0.153, *p* = 0.559). The same held true for the active condition for both the unimodal tactile simulation (r = −0.227, *p* = 0.382) and the audio–tactile one (r = −0.108, *p* = 0.681). As a result, we merged all the blind participants into a single group.

**Figure 2:**
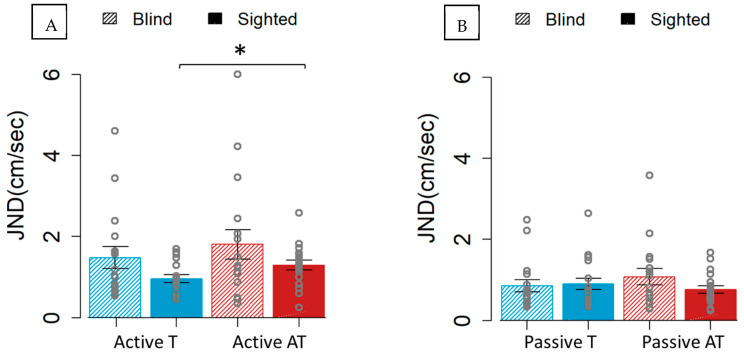
JND for the blind (45° lined boxes) and sighted individuals (full colored boxes) for the two sensory modalities. The blue color represents the tactile (T) sensory stimulus, while the red represents the audio–tactile (AT) sensory stimulus. (**A**) shows the JND obtained in the active condition. (**B**) illustrates the performance in the passive condition. Only the sighted participants had significantly worse precision in the presence of the non-informative sound during active movement when compared to only tactile feedback. * *p* < 0.05.

A worse performance emerged for the audio–tactile than for the tactile sensory stimulus within the active condition for sighted individuals (Z = 0.446, *p* = 0.046) (Figure 2A). Interestingly, this difference was not present in either the passive one (Z = 0.046, *p* = 0.837) (Figure 2B) or in the blind group (active: Z = 0.146, *p* = 0.513; passive: Z = 0.166, *p* = 0.459). These results suggest that only the sighted participants were negatively affected by the non-informative auditory feedback when a movement was involved.

**Figure 3:**
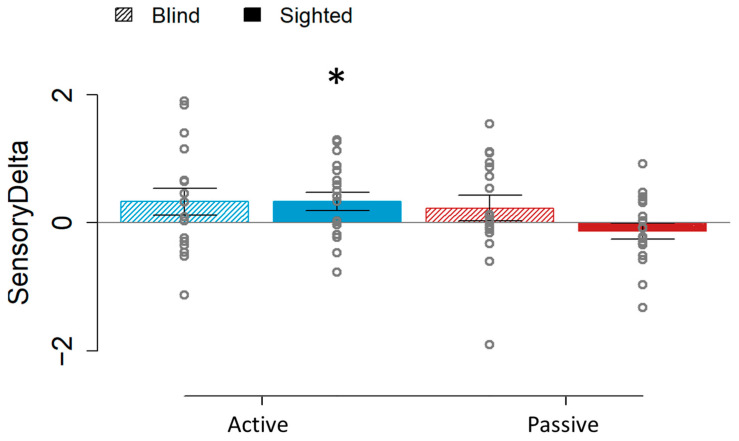
SensoryDelta values for the blind (45° lined boxes) and sighted individuals (full colored boxes) in the active (blue) and passive (red) conditions. Comparing against zero, the difference was significant for the sighted group on the active condition, while it was not for the passive one or for the blind group for any condition. * *p* < 0.05.

To support our results, we computed the SensoryDelta from the audio–tactile and tactile sensory stimulations (Figure 3) for both the active and passive conditions, and we compared it to zero. Here, as we expected, the only significant result was observed in the sighted group on the active condition (t(17) = 2.363, *p* = 0.03), which had a positive delta value (i.e., a higher JND in the bimodal sensory stimulation than in the unimodal one). This indicates that only the sighted participants presented a difference in the audio–tactile performance compared to the tactile one when a self-elicited movement was performed.

**Figure 4:**
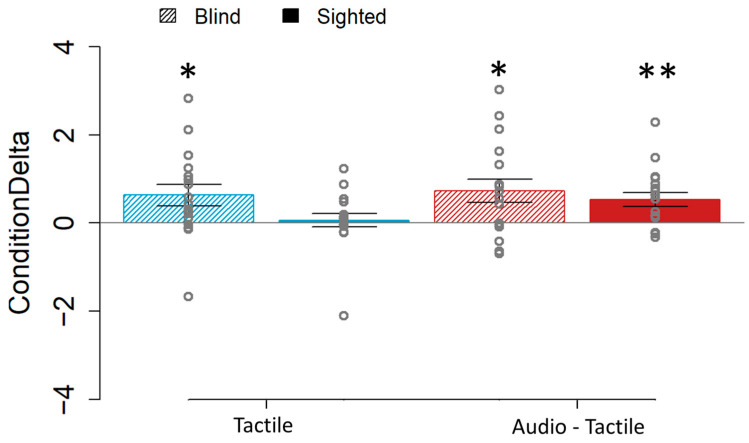
ConditionDelta values for the blind (45° lined boxes) and sighted individuals (full colored boxes) with the tactile (blue) and audio–tactile (red) stimuli. Compared to zero, the difference was significant for the blind group on the tactile and audio–tactile conditions. In the sighted group, it was only significant for the bimodal (audio–tactile) one. * *p* < 0.05. ** *p* < 0.01.

Finally, to have a clearer picture of the implication of the movement in our results, we calculated the ConditionDelta from both the active and the passive conditions, for the tactile and audio–tactile sensory stimulus (Figure 4). In this case, in the blind group, there was a positive delta value (resulting from a higher JND in the active condition than the passive one) that was significantly different from zero for both the bimodal audio–tactile (t(16) = 2.804, *p* = 0.013) and unimodal tactile (t(16) = 2.599, *p* = 0.019). In contrast, in sighted individuals, only the bimodal sensory stimulus had a positive delta value significantly different from zero (audio–tactile: t(17) = 3.31, *p* = 0.004; tactile: Z = 0.268, *p* = 0.2309). These results implied that there is always a significant higher JND in the active conditions in blind individuals. However, in their sighted counterparts, the precision is only negatively affected when the movement is coupled with non-informative feedback.

## 4. Discussion

When the signal from one sensory modality increases its noise, humans combine the information conveyed by different senses to create a more reliable perception of their environment [1]. During active touch, although we know that the movement of body parts can affect the encoding of tactile information [17,31,32], researchers have neglected to study how active movement can influence multisensory processing and the role of vision in this process.

In this study, we found (1) a non-significant difference between the unimodal and the bimodal stimulation for both groups in the passive condition as well as (2) in the active condition for blind participants. However, there were (3) significantly different performances between unimodal tactile stimulation and the bimodal audio–tactile one during active touch in the sighted participants. In other words, the results obtained from this experiment supported our hypotheses.

Using a custom-made auditory device and a physical wheel, we provided dynamic auditory and tactile stimulation on the participant’s index fingers. While discriminating for differences in speed, we measured the precision in sighted individuals in a pure tactile modality (the dataset was obtained from a previous experiment [23]) and in an audio–tactile one. The tactile perception could be either passive, where participants maintained a fixed finger and felt the movement of the rotating wheel, or active, a condition in which participants moved their finger contrary to the wheel’s movement.

Our first result showed that members of the sighted group had worse precision in the presence of the audio–tactile sensory stimulus than in the tactile-only when active movements were involved but not when their perception was static. In contrast, no difference was found in the blind group. To disentangle and support these results, we computed the SensoryDelta from the audio–tactile and tactile conditions (i.e., subtracting the precision obtained in the unimodal condition from the bimodal one) for passive and active touch, separately for blind and sighted participants. In line with the previous findings, there was only a significant positive SensoryDelta value for the sighted group in the active condition. This implies that for this population, the difference between the bimodal and unimodal stimulations was significantly greater than zero, only in the presence of active touch. In other words, our results suggest that, in the sighted group, non-informative auditory inputs only affect their precision when they perform active movements. The movement-related tactile gating might explain this effect. During active touch, the amount of information processed by the cortex can be reduced [18,19], which might be responsible for an increased ambiguity of the tactile signal and internal noise. This has been translated into a higher detection threshold [33,34], worse precision [35], and higher detection rates [22,36] when the body is in motion compared to when it is at rest. At the neurophysiological level, several studies have demonstrated that this effect results from the inhibition of somatosensory evoked potentials during active movement—occurring at both subcortical and cortical levels—compared to rest [18,37]. Internal noise within the brain (i.e., random disturbances in signals) has been proposed as a factor contributing to behavioral variability [38] and driving alternations in perception. Our brain typically tries to solve ambiguity and noise in multiple ways, such as extracting information about the stimulus through different modalities [39]. The stream–bounce illusion provides an example of an ambiguous signal. In this illusion, two stimuli move towards each other until they coincide, a moment in which they change their direction. This could then be interpreted by the viewer as (i) a collision between the two stimuli that induced a bounce in different directions or (ii) both stimuli streaming in different paths. Here, visual feedback does not provide a robust perception. Interestingly, the addition of a sound at the moment of the encounter increased the likelihood of the stimuli bouncing [39]. This is proof that when the signal is ambiguous, our perception is supported by multimodal cues. Parise and Ernst (2017) [1] have also shown using the same illusion that the brain initially processes visual and auditory information separately to extract cues for disambiguation. It then integrates these cues using weighted averaging in a Bayesian manner, forming a coherent perception. They suggest that noise not only triggers perceptual alternation but also systematically biases interpretations toward specific outcomes. Despite with our paradigm we cannot test for multisensory integration, our results show an interaction between tactile and auditory signals coherent with the results found in literature. It suggests that the internal noise generated by movement could induce an interaction between the tactile feedback and the non-informative auditory one, as the brain may anticipate extracting speed information from the temporal patterns of the auditory signal [40]. However, when the signal is sufficiently reliable to provide a robust estimate (e.g., in the passive touch condition), the brain disregards the additional sensory feedback.

Nevertheless, this explanation does not fully apply to the blind group. Indeed, the sound’s presence did not impact the precision of this group, regardless of the type of tactile perception. This supports previous studies that extensively reported a reduced interaction between touch and audition in this population [12,13,16,41]. A successful integration of multisensory information requires to decode between different reference frames and to know the cues that can be merged into a single perception [7]. To achieve this milestone, the cross-modal calibration theory states that the sense that offers the most robust information calibrates and tunes the rest of the sensory modalities, allowing a posterior proper weighting of the sensory signals and, thus, resulting in its integration. As vision, the most relevant sense in spatial perception, is missing in blindness, this tune between senses might fail, weakening multisensory processing and the automatic combination into a common reference frame [13,16].

In addition, to dive into the implications of self-elicited movement in these results, we also calculated the ConditionDelta from the active and passive conditions for both sensory stimuli. Sighted individuals showed a significant positive difference to zero in the bimodal condition but not in the unimodal condition. This suggests that the presence of movement only caused worse precision when it was accompanied by the noise created by the non-informative sound in the control participants. In contrast, the blind group presented significant results in both sensory stimuli, always showing a worse performance in the active condition. In line with our previous work, in which we focused on the effects of self-elicited movement on tactile perception, the significance of the ConditionDelta from the active and passive touch conditions for both the tactile and audio–tactile feedback reflects that, in this population, a possible inefficient integration between the cutaneous, proprioceptive, kinesthetic, and motor cues are responsible for the decreased tactile reliability (see [23] for further details about this phenomenon). Our results further support the altered interaction between senses in this population. Moreover, the absence of performance differences among blind participants, regardless of the onset of blindness, aligns with previous research suggesting that long-term visual deprivation gradually alters neural responses related to spatial representation [42]. This finding indicates that a minimum of 10 years of blindness—the shortest duration in our sample—is sufficient for these processes to be affected.

One potential limitation of this study is the role of movement. Because our task was not highly ecological, the impact of movement in cross-modal interactions in situations where it is relevant remains unclear. For instance, some authors have suggested that perception may be enhanced in such contexts [43]. Additionally, since the velocity of participants’ movements was controlled through instructions and training rather than recorded, possible deviations from the required one may have also influenced our results. Future research should further investigate the role of these factors in the impact of movement on cross-modal interactions.

## 5. Conclusions

In our daily lives, we are exposed to multisensory cues that allow us to create a coherent representation of our environment, with which we relate through our actions. However, the use of active touch in multisensory research is limited, especially in conditions of visual deprivation. Our work shed light on this topic, and suggests that active touch can introduce noise to the sensory signal modulating cross-modal interactions in sighted participants, making them vulnerable to auditory feedback interference. These results highlight the need to select the proper auditory stimulation; non-informative auditory signals might interact with the tactile cues processed during movement and cause a detrimental effect on the tactile perception of this group. However, the precision of blind participants only seems affected by movement and never by non-informative auditory feedback. This finding emphasizes the need for interventions to help this population to (i) enhance somatosensory–motor integration, for instance through games or training sessions including proprioceptive and motor tasks, as well as (ii) promoting the integration of sensory information across different reference frames by activities that align the spatial locations of sounds with tactile perceptions. Overall, these results yield important implications that should be considered in developing next-generation haptic devices and in the rehabilitation field.

## Figures and Tables

**Table 1:** Blind participants’ clinical details, including gender, age, timing of blindness onset, pathology, and remaining visual perception.

Participant	Gender	Age	Blindness Onset	Pathology	Visual Perception
S1	F	22	0	Retinopathy of prematurity	Lights and shadows
S2	F	34	0	Retinopathy of prematurity	No
S3	F	50	0	Retinopathy of prematurity	Lights and shadows
S4	F	32	0	Hereditary retinal dystrophies Retinitis pigmentosa	Lights and shadows
S5	F	32	0	Retinopathy of prematurity	No
S6	M	49	0	Congenital atrophy of the optic nerve	No
S7	M	52	40	Optic nerve subatrophy for a benign tumor	Lights and shadows
S8	M	29	0	Leber Amaurosis	No
S9	F	56	35	Retinitis pigmentosa	Lights and shadows
S10	M	49	6	Congenital glaucoma	No
S11	M	33	17	Corneal opacity	No
S12	M	43	26	Leber Amaurosis Retinitis pigmentosa	Lights and shadows
S13	F	43	0	Atrophy of the optic nerve	No
S14	F	54	0	Retinitis pigmentosa	No
S15	M	59	0	Glaucoma	No
S16	F	56	46	Dystrophies primarily involving the retinal pigment epithelium Leber’s congenital amaurosis	No
S17	M	33	20	Degenerative oculopathy of both eyes	Light and shadows with strong contrast, even colors, a field of view < 1%
S18	F	24	10	Alstrom syndrome Retinitis pigmentosa	Light and shadows

## Data Availability

Raw data are available on the Zenodo repository (10.5281/zenodo.10008441 and 10.5281/zenodo.6581096). The code can be sent upon request. This study can be found as a preprint in the BioRxiv preprint server (https://www.biorxiv.org/content/10.1101/2023.10.30.564759v1, accessed on 1 November 2023).

## Supplementary Materials

The following supporting information can be downloaded at: https://www.mdpi.com/article/10.3390/brainsci15030288/s1. Supplementary file: Normality Assumptions.
