# Supplementary material for: Cross-Modal Interactions and Movement-Related Tactile Gating: The Role of Vision
Source: Brain Sci. 2025 Mar 8;15(3):288. doi: 10.3390/brainsci15030288 (PMC11939845; doi:10.3390/brainsci15030288)
Supplement: Supplementary file 1 [file brainsci-15-00288-s001.zip › brainsci-3450949-supplementary.pdf]

brain sciences

Normality Assumptions

To examine the just noticeable difference (JND) variable, the Jarque Bera test showed that the normality assumption was respected in the sighted group for the active conditions (tactile: X-squared = 1.448, df = 2, p = .485; audio–tactile: X-squared = .652, df = 2, p = .722) and in the passive condition when audio–tactile feedback was provided (X-squared = 2.766, df = 2, p = .251); however, it was not respected for the unimodal tactile presentation (X-squared = 10.297, df = 2, p = .006). For the blind group, the Jarque Bera test indicated a normality assumption violation in the active touch condition (tactile: X-squared = 10.092, df = 2, p = .006; audio–tactile: X-squared = 9.367, df = 2, p = .009) and the passive one (tactile: X-squared = 11.636, df = 2, p = .003; audio–tactile: X-squared = 14.591, df = 2, p = .001).

Regarding the SensoryDelta variable, the Jarque Bera test showed that the normality assumption was respected in the sighted group for both the active condition (X-squared =.601, df = 2, p = .741) and the passive one (X-squared = .324, df = 2, p = .851). It was also respected in the blind group for both the active touch condition (X-squared = .635, df = 2, p = 0.728) and the passive touch condition (X-squared = 3.244, df = 2, p = .198).

Regarding the ConditionDelta variable, the Jarque Bera test showed that the normality assumption was respected in the sighted group for the audio–tactile sensory stimulation (X-squared = 2.307, df = 2, p = .317) but not for the tactile one (X-squared = 358.73, df = 2, p < .001). In the blind group, the normality assumption was always respected (tactile: X-squared = 1.546, df = 2, p = .462; audio–tactile: X-squared = 1.187, df = 2, p = .552).
